# Natural occurrence of pure nano-polycrystalline diamond from impact crater

**DOI:** 10.1038/srep14702

**Published:** 2015-10-01

**Authors:** Hiroaki Ohfuji, Tetsuo Irifune, Konstantin D. Litasov, Tomoharu Yamashita, Futoshi Isobe, Valentin P. Afanasiev, Nikolai P. Pokhilenko

**Affiliations:** 1 Geodynamics Research Center, Ehime University, Matsuyama, Ehime 790-8577, Japan; 2 Earth-Life Science Institute, Tokyo Institute of Technology, Tokyo 152-8550, Japan; 3 V.S. Sobolev Institute of Geology and Mineralogy, Siberian Branch, RAS, Novosibirsk, 630090, Russia; 4 Novosibirsk State University, Novosibirsk, 630090, Russia

## Abstract

Consolidated bodies of polycrystalline diamond with grain sizes less than 100 nm, nano-polycrystalline diamond (NPD), has been experimentally produced by direct conversion of graphite at high pressure and high temperature. NPD has superior hardness, toughness and wear resistance to single-crystalline diamonds because of its peculiar nano-textures, and has been successfully used for industrial and scientific applications. Such sintered nanodiamonds have, however, not been found in natural mantle diamonds. Here we identified natural pure NPD, which was produced by a large meteoritic impact about 35 Ma ago in Russia. The impact diamonds consist of well-sintered equigranular nanocrystals (5–50 nm), similar to synthetic NPD, but with distinct [111] preferred orientation. They formed through the martensitic transformation from single-crystal graphite. Stress-induced local fragmentation of the source graphite and subsequent rapid transformation to diamond in the limited time scale result in multiple diamond nucleation and suppression of the overall grain growth, producing the unique nanocrystalline texture of natural NPD. A huge amount of natural NPD is expected to be present in the Popigai crater, which is potentially important for applications as novel ultra-hard material.

Nano-polycrystalline diamond (NPD)^1^,^2^ synthesized by direct conversion of graphite contributes to the technical innovation in precision machining and fabrication of hard materials^3^. The ultra-high hardness and mechanical strength of NPD arises from the well-sintered nanostructure itself. It prevents the development of micro-cleavage and dislocation movement at grain boundaries, enhancing the bulk strength^4^, as predicted by the Hall-Petch relation^5^,^6^. The production of nano-polycrystalline texture at high P-T is, therefore, a state-of-the-art breakthrough in the development of hard materials and has been applied also for SiO_2_ (stishovite)^7^ and Al_2_O_3_ (corundum)^8^. Here, we identified a natural counterpart of NPD, which has similar microtextures and formation mechanism to those of synthetic one, in diamonds collected from a giant impact crater.

Large meteoritic impact occasionally produces diamond as a result of a shock event on the Earth’s surface^9^,^10^,^11^,^12^,^13^,^14^,^15^,^16^. Popigai crater, located in the north central Siberia, Russia, is one of the major hosts for such impact diamonds^14^,^15^,^16^. It has recently been brought back into the spotlight due to its vast estimated reserves of diamond, although extensive geological exploration of the impact structure and the discovery of impact diamonds were already made in 1970’s^14^,^15^,^16^. Authigenic impact diamonds occur in shocked and fragmented graphite-bearing garnet-biotite gneisses (Archean) that are found as inclusions in impact melt rocks, called tagamites and suevites^14^,^15^,^16^. They occur as irregular to tabular grains of usually 0.5–2 mm size (up to 10 mm) with yellow, gray or black colors and sometimes show notable birefringence^14^,^15^,^16^. The surface of the most impact diamonds displays dissolution and corrosion patterns indicating the experience of intense heating and oxidation in the host impact melt^14^. Earlier studies^14^,^15^,^16^,^17^,^18^ described that they are polycrystalline aggregates of a micron to submicron diamond crystals. The occurrence of apographitic diamond grains (pseudomorphic after single-crystal graphite) and the presence of lonsdaleite, a hexagonal polymorph of diamond (up to 25% of the whole^17^) in most of those grains imply their martensitic formation from well-crystalline graphite. However, in spite of these previous studies, the details of the microtexture and crystallographic features of Popigai diamonds have not been clearly identified (i.e. the overall textural characteristics remained unclear). In most cases, earlier observations by transmission electron microscopy (TEM) observations were made only at local scales on crushed or ion-thinned samples. The present study reveals the nanocrystalline nature of the Popigai impact diamonds through careful TEM observations on a number of oriented cross-sections prepared using focused ion beam (FIB) and discusses the unique transformation and texturing process of the natural NPD.

## Results

**Figure 1:**
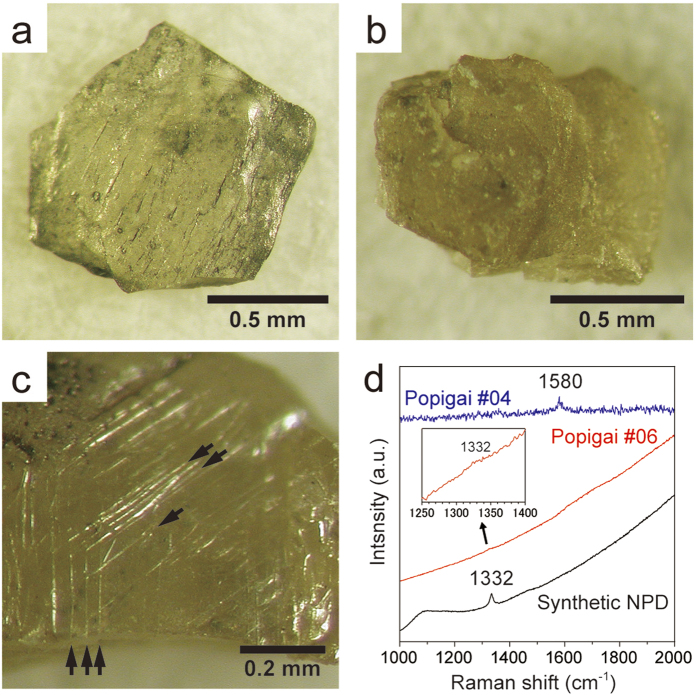
Impact diamonds collected from the Popigai crater. (**a**) Sample #05 composed purely of diamond. (**b**) Sample #08 composed of a mixture of diamond and small amount of lonsdaleite. (**c**) A magnified view of #06 sample showing surface striations intersecting each other at 120° (indicated by arrows). (**d**) Raman spectra of #04 and #06 Popigai diamonds and synthetic NPD (for comparison).

We examined 10 diamond samples separated from tagamites (impact melt rocks) from the Popigai crater. Diamond grains are tabular in shape and 1–1.5 mm wide and 0.1–0.4 mm thick. Seven of them are transparent and show pale-yellow to brownish-yellow colors (Fig. 1a–c), while the other three are partly/fully black and opaque (Fig. S1). Some samples exhibit striations on the surface, which intersect at 120 degrees with each other (Fig. 1c). Such surface features and the tabular morphology imply that single-crystalline graphite is the source material for those diamond grains as noted by previous studies^17^,^18^. Micro Raman analyses showed no detectable peaks (even in the magnified inset), but instead a significant increase (toward higher frequency side) in background intensity due to strong fluorescence (Fig. 1d). Similar background increase is also observed commonly in laboratory-synthesized NPD^19^ and is indicative of the nanocrystalline characteristic of the constituent grains. Opaque samples (#03 and #04) showed broad peaks at 1580 cm^−1^ which can be assigned to E2g stretching mode of graphite.

**Figure 2:**
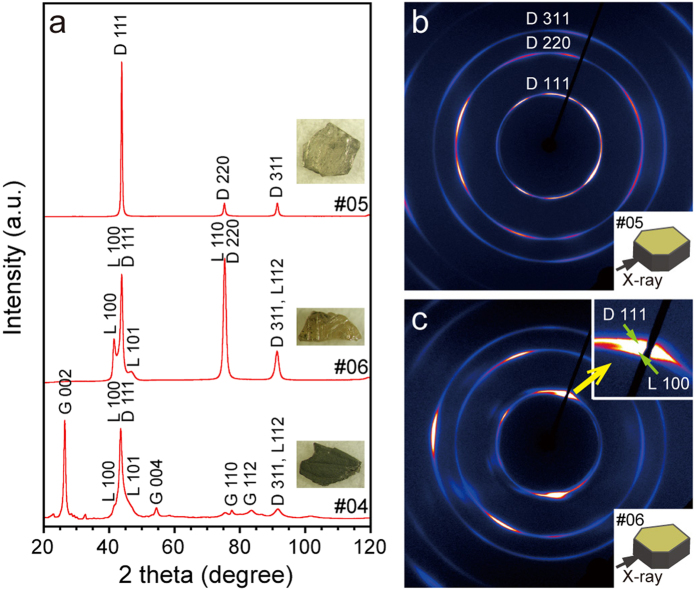
XRD patterns of Popigai impact diamonds. (**a**) XRD spectra collected from black (#04), brownish-yellow (#06) and pale-yellow (#05) impact diamonds in reflection geometry. (**b**,**c**) Two-dimensional XRD patterns collected from #05 and #06 samples, respectively, in transmission geometry. X-ray beam was directed to the tabular samples from the lateral side.

Figure 2a shows typical XRD profiles of Popigai diamond samples with pale-yellow (#05), brownish-yellow (#06) and opaque (#04) colors, measured by a micro-focus XRD in reflection geometry. The transparent samples consist of diamond and occasionally small amounts of lonsdaleite as indicated by small 100 and 101 peaks aside of the diamond 111 peak. The opaque samples consist mostly of diamond and lonsdaleite, but also contain detectable amounts of graphite in agreement with the micro-Raman results. There seems to be a correlation between the sample color and the constituent mineral phases: as the color changes from pale-yellow to brownish-yellow to black, the relative proportion of lonsdaleite and then graphite increases. To investigate the crystallographic orientation and lattice relation between those carbon polymorphs, we further analyzed the samples along the perpendicular directions to the top surface in transmission geometry. Figure 2b,c show typical examples of 2D diffraction patterns (#05 and #06) indicating distinct lattice preferred orientations of the constituent diamond grains. Variable degree of preferred orientations was found in all the samples: many of them are characterized by the high concentrations of diamond 111 in the north-to-south direction of the patterns (Fig. 2c). In the samples containing lonsdaleite ± graphite, concentrations of lonsdaleite 100 were also observed adjacent to the diamond 111 arcs (inset of Fig. 2c) and graphite 002 also shows concentrations along the same directions. These coaxial relations between diamond [111]*, lonsdaleite [100]* and graphite [002]* provide direct evidence for the martensitic formation^20^,^21^,^22^,^23^ of Popigai diamonds from the source graphite via lonsdaleite as an intermediate phase. Other carbon polymorphs such as new transparent cubic phase^24^, which was previously reported from gneisses form the Popigai crater, are not found in the studied samples.

**Figure 3:**
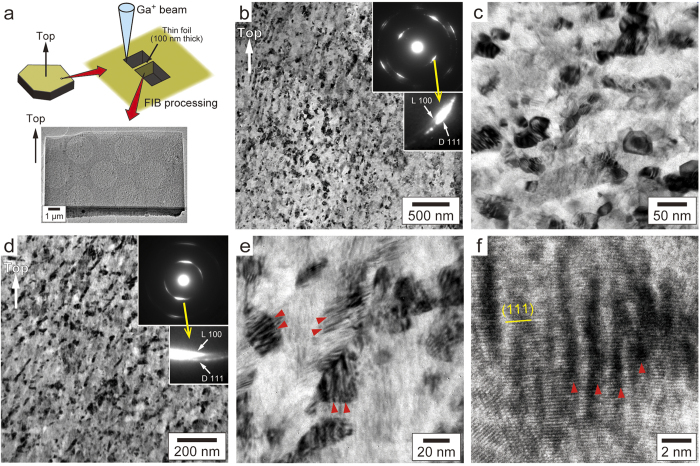
Microtexture of Popigai impact diamonds. (**a**) Schematic illustration of FIB processing. (**b**) Bright-field TEM image and corresponding ED pattern of #05 sample. (**c**) Magnified image of (**b**). (**d**) TEM image and corresponding ED pattern of #06. (**e**) Magnified image of (**d**) showing distinct moiré fringes (indicated by arrows). (**f**) High-resolution TEM image of the grain showing moiré fringes, characteristic of nanoscale rotational deformation feature^25^.

Microtexture of the impact diamonds was examined by TEM on cross-section foils cut out from the top surface of each sample by FIB. The short-side direction of the rectangular foils corresponds to the surface normal of the tabular samples (Fig. 3a). Figure 3b–e show typical examples of TEM images and corresponding selected-area electron diffractions (SAED) of #05 and #06 samples. They are made up of nanocrystals ranging from 5 to 50 nm (mostly 10–20 nm), which tend to be aligned to form a weak lineation (Fig. 3b,d). The SAED patterns display a strong preferred orientation of diamond [111]* being coaxial with lonsdaleite [100]*, which again confirms the martensitic formation of Popigai diamonds from well-crystalline graphite. The preferred orientation direction is tilted against the bottom-top direction of the FIB foil (i.e. sample surface normal) of #05, but almost parallel to that of #06, consistent with the XRD results (Fig. 2b,c). No clear correlations between the preferred orientation (coaxial direction) and weak lineation were found and the origin of lineation is unclear. In magnified images, individual grains frequently show unique moiré fringes (Fig. 2e), created by nanoscale rotational deformation^25^ of the diamond lattices. The origin of such lattice strains might be stacking faults caused by the intercalation of diamond and lonsdaleite layers within the individual grains, because the moirés are less observed in the samples containing less lonsdaleite. Another possibility is the presence of repeated microtwins on diamond (111) planes, which have also been found in Popigai diamonds by earlier studies^26^.

## Discussion

The nanocrystalline texture of Popigai diamonds is well comparable to that of typical laboratory-made NPD^1^,^4^,^27^. This implies that the basics of their crystallization and texturing process are common, although the time scales of the diamond-forming events (dynamic and static, respectively) are different by several orders of magnitudes. Two mechanisms are known to responsible for graphite-diamond transformation under high pressure: martensite transformation and nucleation and growth process^4^,^21^,^27^. The former is a diffusion-less process where diamond formation occurs by the buckling of the basal planes of graphite. Such formed diamonds are often accompanied by lonsdaleite as an intermediate product, and unique crystallographic relationships are found between the source graphite (G), lonsdaleite (L) and diamond (D): (001)_G_//(100)_L_//(111)_D_, [210]_G_//[001]_L_//[2–1–1]_D_ and (1–20)_G_//(−120)_L_//(0–22)_D_^20^,^21^,^23^. On the other hand, diamond formation by the latter mechanism proceeds through diffusion-controlled nucleation and subsequent crystal growth, which initiate preferentially at lattice defects and crystal surfaces where sp^3^-hybridized dangling bounds are dominated^21^,^27^. Experimental studies suggest that the most important and essential factor determining which transformation mechanism is preferable is the crystallinity of initial graphite sources^21^,^27^,^28^,^29^. The martensitic transformation is favored when using well-crystalline graphite as a source material, while the nucleation and growth mechanism becomes dominant when using poor-crystalline, disordered graphite. According to the results of static and shock high-pressure experimental studies^21^,^27^,^28^,^29^, the transformation mechanisms are not influenced by the manner of compression – either statistic or dynamic, and the degree of graphite-diamond transformation is proportional to the magnitude of the thermal effect.

Although Popigai diamonds and synthetic NPD both consist of granular crystals of a few to several tens nanometer, there is a clear difference in crystallographic orientation: the former show a distinct [111] preferred orientation, while the latter shows basically a random orientation (Fig. S2a). This is probably due to the differences in crystalline nature of the initial graphite sources. The source materials for Popigai impact diamonds and NPD are single-crystalline graphite and a dense compact of nanocrystalline graphite^27^, respectively. Therefore, the diamond formation from the former occurs predominantly by the martensitic mechanism, while that from the latter occurs by the nucleation and growth mechanism. Diamonds produced by the martensitic process usually show a layered texture with a preferred orientation of diamond [111] along the stacking direction (c-axis) of the source graphite^20^,^21^,^27^,^28^. A typical example is layered nanodiamonds recently synthesized from highly oriented pyrolitic graphite (HOPG) at HPHT^29^ (Fig. S2b). However, despite showing the distinct [111] preferred orientation, Popigai diamonds seldom contain lamellar or layered crystals (although granular crystals are often aligned to form weak lineations (Fig. 3d), they seem to be not associated with the preferred orientation). This can also be attributed to the crystalline feature of the source graphite.

**Figure 4:**
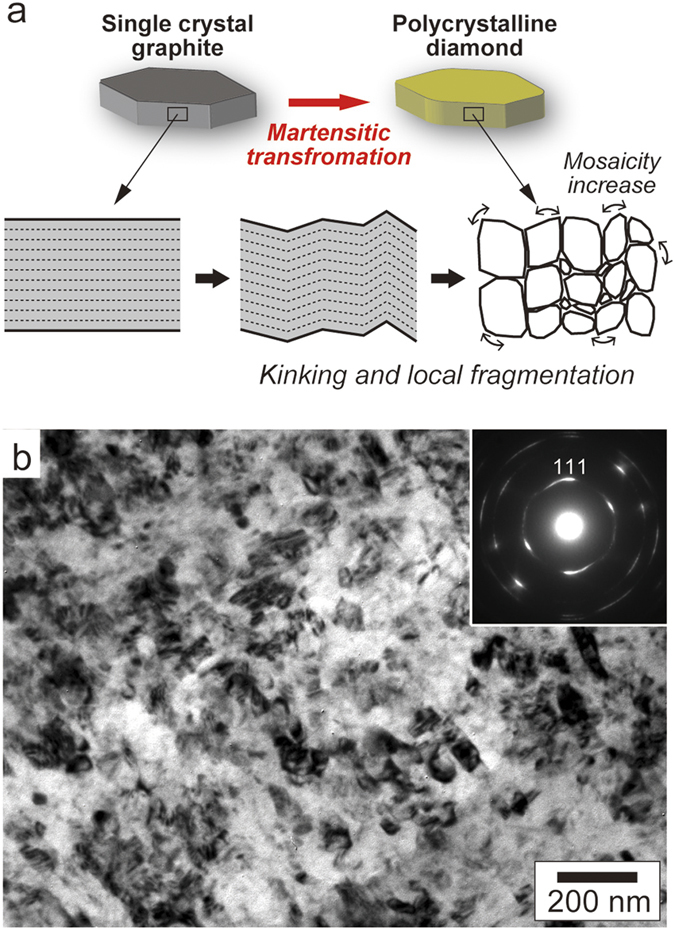
Nanodiamond formation from single crystal graphite. (**a**) Schematic illustration of the formation of mosaic texture through martensitic transformation from single crystal graphite to diamond. (**b**) TEM image and corresponding ED pattern of nano-polycrystalline diamond synthesized from single crystal (Kish) graphite at 15 GPa, 2300 °C.

HOPG, the starting material of the synthetic layered nanodiamonds^29^, consists of graphite tiles of typically 50–100 nm thick, which are all highly oriented along the c-axis (stacking direction) but are randomly oriented along the perpendicular direction. The individual tiles are, therefore, separated with each other by grain boundaries. When it is compressed, the stress accumulated during compression can be relaxed mostly by grain boundary (layer) sliding, and consequently the initial layered texture is preserved after the transformation to diamond upon heating (Fig. S2b). This is, however, not the case for single crystalline graphite, from which the Popigai impact diamonds have been formed. Although a graphite single crystal has a perfect cleavage on (001) at the ambient condition, the interlayer bonding strength increases significantly due to the formation of σ-bonds with increasing pressure^30^. This means that a graphite single crystal (with virtually no grain boundaries) is unlikely to deform by layer sliding. Alternatively, the stress accumulated during compression can be relaxed by the fragmentation (plastic deformation) of the crystal at local scales (Fig. 4a). The mosaic (granular) texture and the apparently larger misorientations of the constituent grains (compared with those of the layered diamonds^21^,^29^) of Popigai diamonds might be evidence of such a deformation process. In fact, shock-induced deformation (kinking) and fracturing of crystalline graphite was observed at the initial stage of the diamond formation in shock compression experiments^31^,^32^. This process results in the formation of a large number of structural defects (i.e. dangling bonds) in graphite, providing preferential nucleation sites for diamond.

To confirm this hypothesis, we performed high pressure experiments using single-crystalline (Kish) graphite as the starting material and attempted to reproduce the unique microtexture of Popigai diamonds. The sample synthesized at 15 GPa and 2300 °C consists mostly of diamond with a trace amount of lonsdaleite and shows a granular texture with a distinct [111] preferred orientation along the [001] of the source graphite (Fig. 4b). The texture is well comparable to that of Popigai diamonds and therefore represents an intrinsic property of the nanodiamond crystalized from single-crystalline graphite. Our experiments also demonstrated that the kinetics for graphite-diamond transformation via intermediate lonsdaleite depends largely on temperature at a fixed pressure: graphite (unreacted residue) and lonsdaleite (intermediate product) are clearly dominant in the products from lower temperatures. Similar kinetic effects were also found in the diamond formation from polycrystalline graphite^2^ and HOPG^29^. This implies that the variety of the phase (polymorphic) compositions in Popigai diamonds (Figs 1a and 2a) is likely due to temperature heterogeneity in the host rocks during the shock event.

Diamond formation through shock-compression of graphite is observed also in some carbonaceous^23^,^33^ and iron meteorites^34^. However, such meteoritic diamonds usually occur at the nano- to micrometer scales as a mixture with the source graphite and metastable lonsdaleite. The coexistence of the carbon polymorphs with certain crystallographic relationships^20^,^21^,^22^,^23^ between their lattices indicates that the diamonds were produced by the martensitic transformation form crystalline graphite upon collision(s) of the host meteorite against other(s)^23^,^33^,^34^. In this respect, the formation process of the impact-produced diamonds from meteorites and meteoritic craters (Popigai) is essentially comparable. However, the unique nano-polycrystalline texture such as shown in Fig. 3 has not been found so far in meteoritic diamonds. This is probably because of the lower magnitude of the impacts; the shock pressure and (more importantly) shock temperature were not sufficient to complete the diamond transformation and to sinter individual diamond crystals. This, however, means that NPD might be found also in meteorites themselves, if the meteorites experienced severe shock event(s) that gave PT conditions exceeding the threshold.

Meteoritic diamonds are known to occasionally contain other metastable carbon polymorphs such as n-diamond^23^, which is proposed to have a face-centered cubic structure with space group Fm3m, showing additional reflections forbidden for diamond^35^,^36^,^37^. The formation of n-diamond is also observed in shock- and static high pressure experiments^35^,^36^ on crystalline graphite. However, despite careful analysis by X-ray and electron diffraction techniques, such intermediate phases were not found in the 10 Popigai diamond samples (even in those containing a larger amount of residual graphite) studied here.

In the present study, we identified the nature of the nanocrystalline texture of Popigai impact diamonds as a natural counterpart of synthetic NPD. We clearly demonstrated the unique texturing process by comparing to the results of high-pressure experiments, which also provides insight in understanding the crystallization mechanism of impact diamonds from other craters^9^,^10^,^11^ and ejecta layers^12^,^13^. The essential requirements for NPD formation in nature can be summarized: 1) abundance of pure carbon sources, and 2) short reaction time under adequate pressure and temperature. In particular, the second point is critical for the nanocrystalline texture to be preserved without further grain growth and recrystallization. Although polycrystalline diamonds, such as carbonado^38^ and ballas^39^, can be found among mantle diamonds, they are aggregates of diamond grains of several tens to hundreds μm. This is simply because of the longer (geological) time scale of the crystallization process in the deep Earth. The Popigai crater satisfactorily provided a suitable condition for NPD formation; graphite crystals were abundantly supplied from the host Archean gneisses^14^, and the impact event converted such graphite crystals into nanodiamonds instantly. The estimated diamond reserves in Popigai crater may reach up to trillions of carats^40^. Since Popigai diamonds possesses a well-sintered, nano-polycrystalline texture comparable to that of laboratory-made NPD, they might be a promising source for industrial applications as ultra-hard tools. Indeed, abrasive property of Popigai diamonds was found to be 1.5–2.0 times higher than that of single crystalline diamond^41^.

## Methods

We studied 10 impact diamond samples separated from tagamite (impact melt rock) that were collected from Skalnoe deposit (71°30′19′′N, 110°23′52′′E) inside the Popigai crater. The original tagamite samples were crushed for 1 mm fraction and heavy concentrate was extracted by flotation method. The concentrate contains 5,000–10,000 carat of diamond per ton. The heavy concentrate, which includes diamonds and other minerals such as garnet, zircon, etc., was separated from the light fraction in bromoform. Finally diamond grains were extracted from the heavy concentrate by Clerici solutions. The diamond samples were examined by Optical microscopic observation, Raman spectroscopy, micro-focused XRD and TEM. Raman spectroscopic measurements were conducted by using a confocal micro-Raman system (Renishaw RS-SYS 1000) and an Ar^+^ laser. XRD measurements were performed by using a micro-focused XRD system (Rigaku Rapid IV) with Mo*Kα* radiation (*λ* = 0.7107 Å, 50 kV, 24 mA). The X-ray diffraction data were collected on a curved imaging plate (film distance: 127.16 mm). For phase identification, the measurements were conducted in reflection geometry with a fixed omega (beam-sample surface) angle of 20 degrees. The 1D profiles were obtained by integrating the intensity of the 2D diffraction patterns over definite angular sectors. The samples were also measured in transmission geometry to investigate the lattice preferred orientation of the constituent crystals. For TEM observations, cross-section foils with a dimension of ca. 12 × 7 × 0.1 μm were cut out from the top surface of the samples using a focused ion beam (FIB) system (JEOL JEM-9310FIB). The TEM observations were conducted using JEOL JEM-2010 operated at 200 kV.

High pressure and high temperature synthesis of diamond from single-crystalline (Kish) graphite was conducted using a 3000 ton multianvil apparatus and 36 mm tungsten carbide anvils with a truncation edge length of 5 mm. The detail of the cell assembly used was described in our previous report^29^. The generated pressure was estimated from a pressure-load calibration curve obtained based on the phase transition of pressure standard materials (ZnTe and ZnS). Temperature was estimated from the relationship between input electrical power and generated temperature obtained in a separate run using the same cell assembly. The sample was compressed to 15 GPa and heated to 1600 and 2300 °C for 20 min.

## Additional Information

**How to cite this article**: Ohfuji, H. *et al.* Natural occurrence of pure nano-polycrystalline diamond from impact crater. *Sci. Rep.* **5**, 14702; doi: 10.1038/srep14702 (2015).

## Supplementary Material

Supplementary Information
