# Supplementary material for: Natural occurrence of pure nano-polycrystalline diamond from impact crater
Source: Sci Rep. 2015 Oct 1;5:14702. doi: 10.1038/srep14702 (PMC4589680; doi:10.1038/srep14702)
Supplement: Supplementary Information [file srep14702-s1.pdf]

Supplementary Information

Natural occurrence of pure nano-polycrystalline diamond from impact crater

*Hiroaki Ohfuji[1], Tetsuo Irifune[1,2], Konstantin D. Litasov[3,4], Tomoharu Yamashita[1], Futoshi Isobe[1], Valentin P. Afanasiev[3] & Nikolai P. Pokhilenko[3]

Affiliations:

[1]Geodynamics Research Center, Ehime University, Matsuyama, Ehime 790-8577, Japan

[2]Earth-Life Science Institute, Tokyo Institute of Technology, Tokyo 152-8550, Japan

[3]V.S. Sobolev Institute of Geology and Mineralogy, Siberian Branch, RAS, Novosibirsk, 630090, Russia

[4]Novosibirsk State University, Novosibirsk, 630090, Russia

***Corresponding author:** Hiroaki Ohfuji ohfuji@sci.ehime-u.ac.jp

This file includes:

Supplementary Figures S1 to S2

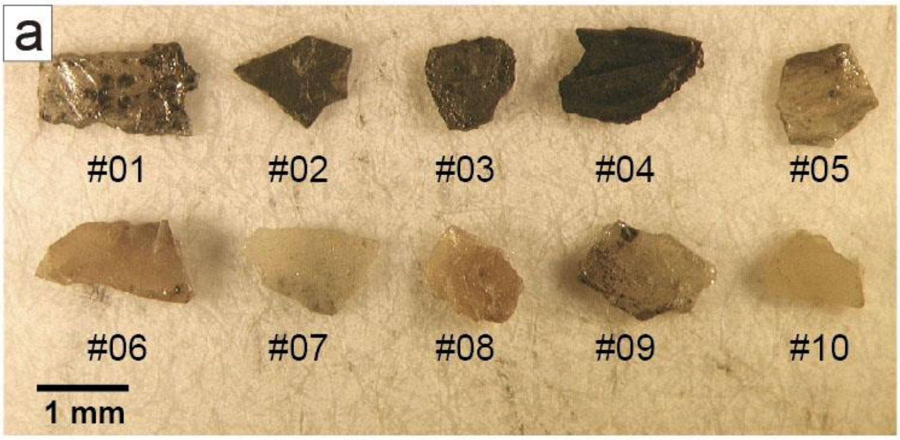

Supplementary Figure S1 | Optical microscopic images of 10 impact diamonds from the Popigai crater. Many of them are transparent and show pale yellowish to brownish yellow colors except for #02, #03 and #04 which are fully or partly opaque.

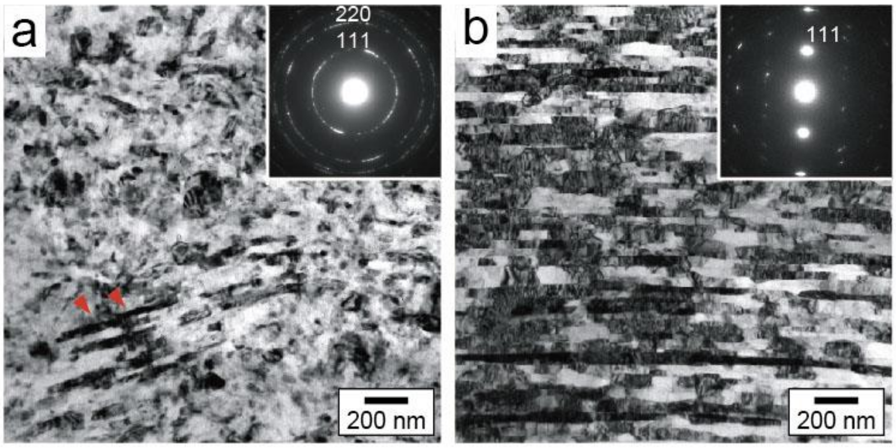

Supplementary Figure S2 | TEM images of nanocrystalline diamonds synthesized by direct conversion of graphite. (a) Typical nano-polycrystalline diamond obtained from polycrystalline graphite, which partially contains lamellar crystals (arrows)[27]. (b) Nano-layered diamond synthesized from highly oriented pyrolitic graphite, showing distinct [111] preferred orientation along the stacking direction [29].
